# Impact of Age on Mortality and Decompensation Events in Patients With Liver Cirrhosis: A Multicenter, Propensity Score Matched Study

**DOI:** 10.1155/ijh/8852224

**Published:** 2025-11-24

**Authors:** Muhammad Shabbir, Miguel Salazar, Zeid Kayali

**Affiliations:** ^1^Department of Gastroenterology and Hepatology, University of California, Riverside, California, USA; ^2^Inland Empire Clinical Trials, Rialto, California, USA

**Keywords:** aging, cirrhosis, decompensation events, mortality

## Abstract

**Background:**

The incidence of cirrhosis is increasing in the older population. Limited data are available on the disease progression and mortality in the older population with cirrhosis. This study is aimed at evaluating the impact of age at diagnosis on all-cause mortality and decompensation events in patients with liver cirrhosis.

**Methods:**

This is a retrospective cohort study utilizing TriNetX. ICD codes were used to identify individuals with the diagnosis of liver cirrhosis between the ages of 20 and 80. Patients with the diagnosis of congestive heart failure (CHF), end-stage renal disease (ESRD), chronic kidney disease (CKD) Stage IV and V, human immunodeficiency virus (HIV), malignant neoplasm, and psychoactive substance abuse were excluded from the analyses. Patients were divided into two cohorts: Cohort 1 included individuals with the diagnosis of liver cirrhosis between the ages of 51 and 80, and Cohort 2 included individuals with the diagnosis between the ages of 20 and 50. Statistical analyses were conducted using TriNetX Live. A 1:1 propensity score matching was performed for variables including race, gender, ethnicity, comorbidities, laboratory values for MELD 3.0, and etiology of liver cirrhosis. There were 70,983 patients in each cohort after matching. The primary outcome was all-cause mortality, and the composite outcome of decompensation events at 5- and 10-year intervals from the age of diagnosis of liver cirrhosis. Secondary outcomes included the risk of decompensation events, all-cause hospitalization at 5-year intervals, and a subgroup analysis of all-cause mortality and decompensation events among males and females.

**Results:**

Older age at diagnosis of liver cirrhosis was associated with increased all-cause mortality at 5 years (aOR 1.378, 95% CI: 1.335–1.422; *p* < 0.001) and 10 years (aOR 1.418, 95% CI: 1.376–1.462; *p* < 0.001). These patients also demonstrated an increased risk of decompensation events at 5 years (aOR 1.236, 95% CI: 1.199, 1.275; *p* < 0.001) and at a 10-year interval (aOR 1.266, 95% CI: 1.229, 1.305; *p* < 0.001). At 5-year intervals, these patients (Cohort 1) were found to have an increased risk of variceal bleeding (aOR 1.309, 95% CI: 1.258–1.361; *p* < 0.001), ascites (aOR 1.114, 95% CI: 1.052–1.180; *p* < 0.001), hepatic encephalopathy (aOR 1.1, 95% CI: 1.026–1.180; *p* < 0.001), hepatopulmonary syndrome (aOR 1.45, 95% CI: 0.820–2.564; *p* = 0.101), and hepatocellular carcinoma (aOR 2.924, 95% CI: 2.477–3.453, *p* < 0.001). Conversely, in younger patients, there were increased odds of developing spontaneous bacterial peritonitis (SBP) (aOR 0.848, 95% CI: 0.720–0.998, *p* = 0.02) and hepatorenal syndrome (HRS) (aOR 0.753, 95% CI: 0.651–0.871, *p* < 0.01). The differences were persistent in a subgroup analysis among males (mortality, aOR 1.37, 95% CI: 1.319, 1.424; *p* < 0.001) and females (mortality aOR 1.384, 95% CI: 1.311, 1.462; *p* < 0.001).

**Conclusion:**

Older age at diagnosis of liver cirrhosis is associated with increased all-cause mortality and key decompensation events. Certain conditions, like SBP and HRS, are more common in the younger population, likely due to increased alcohol abuse. Early detection of portal hypertension and early appropriate prophylaxis for variceal bleeding can provide benefit in this high-risk population, although the exact impact of such strategies needs further studies. Besides early recognition, alcohol remains a key factor that needs to be concomitantly addressed as it drives life-threatening decompensating events.

## 1. Introduction

The global demographic shift toward an aging population has significant implications for chronic liver disease management, particularly cirrhosis. As life expectancy increases due to advancements in healthcare, social, and economic development, the prevalence of cirrhosis is expected to rise in the elderly population [1, 2].

Cirrhosis remains a major global health concern. Even though age-standardized mortality rates have stabilized, the absolute number of cirrhosis cases and deaths continues to rise [3]. The incidence of cirrhosis in elderly patients is expected to increase due to the increase in prevalence of metabolic dysfunction–associated liver disease (MASLD) [4].

Aging leads to important physiologic changes in the liver, including a reduction in liver blood flow and volume. This leads to reduced regenerative potential and weakened intrahepatic immunity due to decreased function of Kupffer cells [5–7]. Survival outcomes differ between age groups, with older patients generally exhibiting poorer survival due to frailty, multiorgan failure, and limited eligibility for liver transplants [8].

This has been attributed to the fact that elderly patients are not usually candidates for liver transplantation, either due to age or related severe comorbidities, and they have poor tolerance to the treatment of decompensation, specifically ascites and hepatic encephalopathy (HE) [9–11]. Previous studies assessing the impact of aging on clinical outcomes of cirrhosis in elderly patients had certain limitations, including single-center designs, small sample size, utilizing a limited number of variables that might impact survival, and a lack of population diversity. To address these limitations, we conducted a multicenter, large-scale study with a diverse population utilizing real-world data.

This study is aimed at evaluating the impact of age at diagnosis on all-cause mortality and decompensation events in patients with liver cirrhosis. By understanding how age influences the incidence and progression of these complications, the study will provide insights into the challenges of managing this growing population of aging patients with cirrhosis. Furthermore, it will provide direction for future research aimed at refining treatment approaches for both younger and older patients with liver disease.

## 2. Study Design

The primary endpoint was all-cause mortality and the composite outcome of decompensation events at 5-year and 10-year intervals from the age of diagnosis of liver cirrhosis among the two cohorts. Cohort 1 included individuals with the diagnosis of liver cirrhosis between the age of 51 and 80, and Cohort 2 included individuals with the diagnosis of liver cirrhosis between the age of 20 and 50. Secondary end points included the odds of decompensation events, including esophageal varices with bleeding, ascites, spontaneous bacterial peritonitis (SBP), hepatorenal syndrome (HRS), hepatopulmonary syndrome (HPS), HE, risk of hospitalization, and hepatocellular carcinoma (HCC) during the 5-year interval, and a subgroup analysis among males and females.

## 3. Methods

TriNetX, a global health research network, provided access to electronic medical records (diagnoses, procedures, medications, laboratory values, and genomic information) from approximately one hundred million patients from 141 healthcare organizations (HCOs).

This study was performed on August 08, 2025, on the global collaborative network. All data collection, processing, and transmission were done in compliance with all Data Protection laws applicable to the contributing HCOs, including the European Union's (EU) General Data Protection Regulation 2016/679, which protects the processing of personal data, and the US federal laws, specifically the Health Insurance Portability and Accountability Act, which protects the privacy and security of healthcare data.

TriNetX is International Organization for Standardization (ISO) 27001:2013 certified and maintains a robust IT security program that protects both personal data and healthcare data. The analysis was done with TriNetX LIVE built-in analytics.

The global collaborative network is a distributed network, and analytics are performed at the HCO, with only aggregate results being returned to the platform. *International Classification of Disease, Tenth Revision, Clinical Modification* (*ICD-10-CM*) codes were used to identify cohorts.

Inclusion criteria included patients with the diagnosis of liver cirrhosis with an age at diagnosis between 20 and 80. Patients with the diagnosis of ESRD, CKD Stage IV and V, heart failure, malignant neoplasm, HIV, and psychoactive substance abuse were excluded from the study. In addition, patients with any decompensation event prior to the onset of liver cirrhosis were excluded from the analysis (Table S1).

The total pool meeting the above criteria was further subdivided into two cohorts. Cohort 1 included individuals with a diagnosis of liver cirrhosis with the age at diagnosis between 51 and 80, and Cohort 2 included individuals with the age at diagnosis between 20 and 50.

The index event for both groups was defined as the onset of liver cirrhosis. To avoid overlap between the two groups and to ensure that the groups are mutually exclusive, patients with a diagnosis of liver cirrhosis between the ages of 20 and 50 were excluded from Cohort 1 and vice versa.

The most common etiology of liver cirrhosis was fatty liver (10.3% vs. 10.1%, *p* = 0.194), followed by chronic viral hepatitis C (4.5% vs. 4.5%, *p* = 0.759) and alcoholic liver disease (4.2% vs. 4.2%, *p* = 0.50).

**Figure 1:**
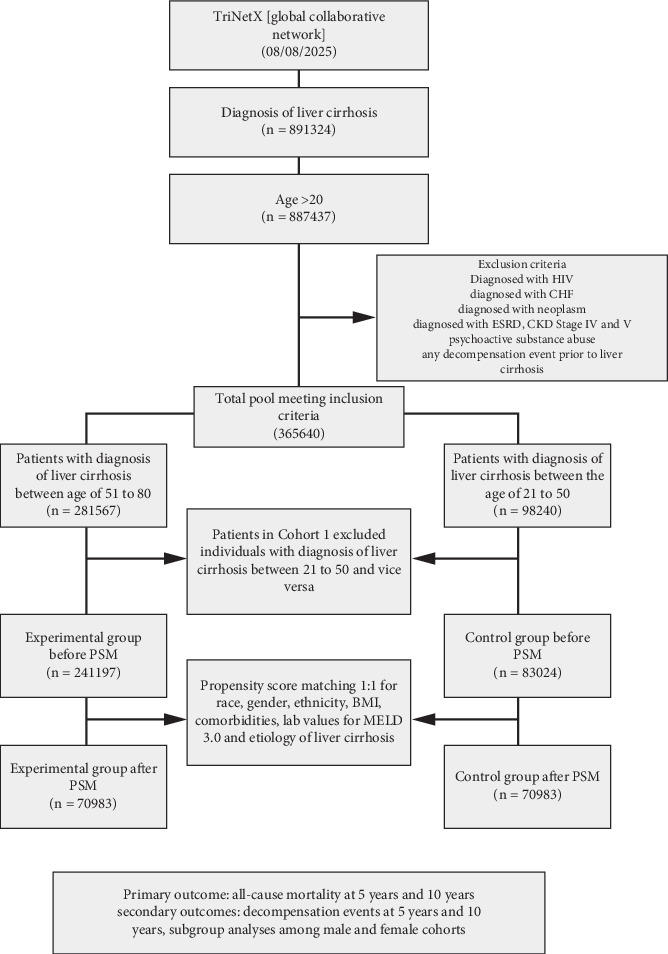
Age at diagnosis is a predictor of mortality in patients with liver cirrhosis.

A flow chart diagram with the inclusion and exclusion criteria is shown in Figure 1. The primary endpoint, all-cause mortality, is determined when the patient becomes deceased in the TriNetX network. The platform tests for mortality by various methods, including a recorded death event in the electronic health record (EHR) a discharge code indicating death. Secondary outcomes were assessed using the ICD-10 codes (Table S2).

This study is exempt from informed consent. The data reviewed is a secondary analysis of existing data, does not involve intervention or interaction with human subjects, and is deidentified per the deidentification standard defined in Section §164.514(a) of the HIPAA Privacy Rule. The process by which the data is deidentified is attested to through a formal determination by a qualified expert as defined in Section §164.514(b)(1) of the HIPAA Privacy Rule.

## 4. Statistical Analysis

All statistical analyses were performed using TriNetX through its browser-based real-time analytics platform, TriNetX Live (TriNetX LLC). The baseline characteristics of both cohorts were defined using the mean and standard deviation (SD). One-to-one (1:1) propensity score matching (PSM) was performed for gender, race, ethnicity, BMI, diabetes mellitus (DM), ischemic heart disease, other forms of heart disease, cerebral infarction, chronic lower respiratory disease, hypertensive disorders, peripheral vascular disease, lab values for MELD 3.0 (serum bilirubin, albumin, INR, serum sodium, and serum creatinine), fatty liver, alcoholic liver disease, toxic liver disease, and chronic viral hepatitis B and C. These variables were selected because they have been found to impact survival in patients with liver cirrhosis. PSM was performed using the TriNetX platform, which applies logistic regression to estimate propensity scores based on user-defined covariates. Matching was conducted using a greedy nearest-neighbor algorithm with a caliper width of 0.1 pooled SDs. To minimize selection bias, the platform randomizes the order of rows prior to matching. Postmatching *p* values assess the balance of covariates between cohorts. The *t*-test was used to compare numerical lab values, and the chi-square was used to compare categorical lab values. Following matching, outcomes were analyzed using adjusted odds ratios (aORs) with 95% confidence intervals (CI). Two-sided *p* values less than 0.05 were considered statistically significant. The platform uses R's survival package v3.2-3 to calculate the hazard ratio (HR).

## 5. Results

**Figure 2:**
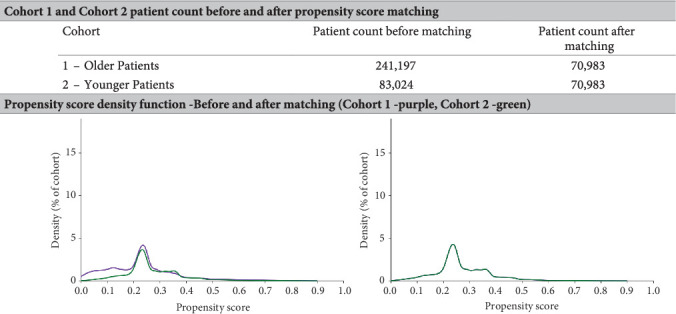
Graph before and after propensity score matching.

There were 70,983 patients in each cohort after PSM (Figure 2). A table of baseline demographics, comorbidities, etiology of liver cirrhosis, and means of laboratory values is shown in Table 1.

Mean current age of Cohort 1 was 69.1 ± 8 years compared to 47.9 ± 9 years in Cohort 2. Mean age at index event was 62.4 ± 7.4 years versus 40.9 ± 7.3 years, respectively (*p* < 0.001). The majority of the patients were White (58.3% vs. 58.0%, *p* = 0.186) and African American (6.5% vs. 6.9%, *p* = 0.001). Male patients had a higher proportion than females in both cohorts (61.5% vs. 61.4% male, *p* = 0.731; 38.5% vs. 38.6% females, *p* = 0.756). Among the ethnicities, the majority were non-Hispanic (53.2%, *p* = 0.836), Hispanic (12.5%, *p* = 0.974), and Asian (9.4% vs. 8.9%, *p* = 0.002).

A significant proportion of patients had DM (9.4% vs. 9.4%, *p* = 0.993) and chronic lower respiratory diseases (7.6% vs. 7.6%, *p* = 0.609). The proportion of ischemic heart disease and other forms of heart disease was 2% (*p* = 0.317) and 6% (*p* = 0.717), respectively, in both cohorts.

The most common etiology of liver cirrhosis was fatty liver (10.3% vs. 10.1%, *p* = 0.194), followed by chronic viral hepatitis C (4.5% vs. 4.5%, *p* = 0.759). Mean MELD 3.0 at baseline was 10 for males and 12 for females, for both cohorts. Mean MELD 3.0 at a 10-year interval was 20 in males and 21 in females in Cohort 1 and 19 in males and 20 in females in Cohort 2 (Table 2). Mean laboratory values of both cohorts at 5- and 10-year intervals are shown in Tables 3 and 4, respectively (Table S3).

Older age at diagnosis of liver cirrhosis (Cohort 1) was associated with increased all-cause mortality at 5 years (aOR 1.378, 95% CI: 1.335, 1.422; *p* < 0.001) and 10 years (aOR 1.418, 95% CI: 1.376, 1.462; *p* < 0.001) (Tables 5 and 6). These patients also demonstrated increased odds of decompensation events (composite outcome) at 5 years (aOR 1.236, 95% CI: 1.199, 1.275; *p* < 0.001) and 10 years (aOR 1.266, 95% CI: 1.229, 1.305; *p* < 0.001).

**Figure 3:**
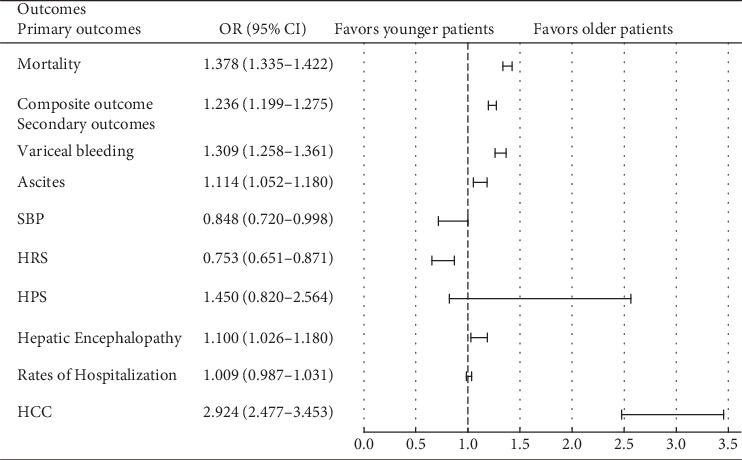
Outcomes at 5-year interval among older versus younger cirrhotic patients. SBP, spontaneous bacterial peritonitis; HRS, hepatorenal syndrome; HPS, hepatopulmonary syndrome; HE, hepatic encephalopathy; HCC, hepatocellular carcinoma.

At a 5-year interval, Cohort 1 was found to have increased risk of variceal bleeding (aOR 1.309, 95% CI: 1.258–1.361, *p* < 0.001), ascites (aOR 1.114, 95% CI: 1.052–1.180, *p* < 0.001), HE (aOR 1.1, 95% CI: 1.026–1.180, *p* < 0.001), and HPS (aOR 1.45, 95% CI: 0.820–2.564, *p* = 0.10). The odds of developing HCC were markedly elevated in older patients (aOR 2.924, 95% CI: 2.477–3.453, *p* < 0.001), indicating a strong predisposition of age at index event to malignant transformation. Conversely, in younger patients, there were increased odds of developing SBP (aOR 0.848, 95% CI: 0.720–0.998, *p* = 0.02) and HRS (aOR 0.753, 95% CI: 0.651–0.871; *p* < 0.001) (Table 5 and Figure 3).

**Figure 4:**
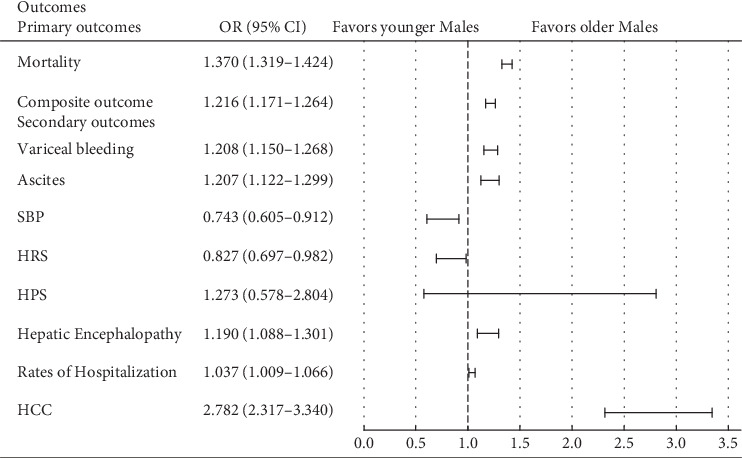
Outcomes at 5-year interval among older versus younger males. SBP, spontaneous bacterial peritonitis; HRS, hepatorenal syndrome; HPS, hepatopulmonary syndrome; HE, hepatic encephalopathy; HCC, hepatocellular carcinoma.

**Figure 5:**
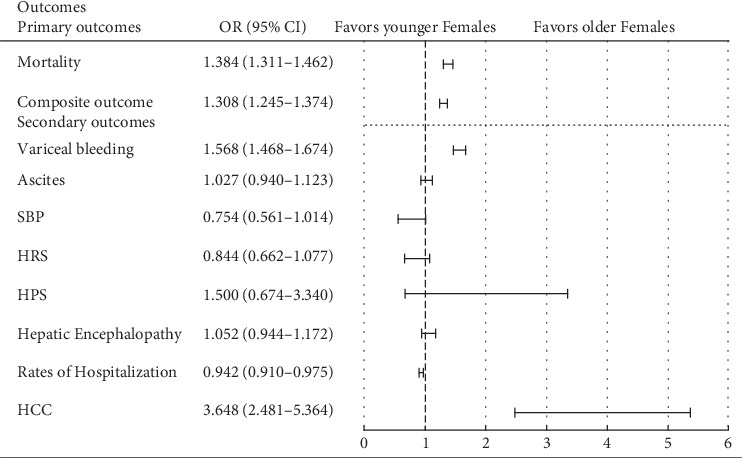
Outcomes at 5-year interval among older versus younger females. SBP, spontaneous bacterial peritonitis; HRS, hepatorenal syndrome; HPS, hepatopulmonary syndrome; HE, hepatic encephalopathy; HCC, hepatocellular carcinoma.

In a subgroup analysis, among older versus younger male patients, the differences were persistent at 5 years, including all-cause mortality (aOR 1.370, 95% CI: 1.319–1.424; *p* < 0.001), composite outcome of decompensation events (aOR 1.216, 95% CI: 1.171–1.264; *p* < 0.001), variceal bleeding (aOR 1.208, 95% CI: 1.150, 1.268; *p* < 0.001), ascites (aOR 1.207, 95% CI: 1.122, 1.299; *p* < 0.001), HE (aOR 1.19, 95% CI: 1.088, 1.301; *p* < 0.001), and HCC (aOR 2.782, 95% CI: 2.317, 3.340; *p* < 0.001). Cohort 2 (younger males) had increased odds of HRS (aOR 0.827, 95% CI: 0.697, 0.982; *p* = 0.015) and SBP (aOR 0.743, 95% CI: 0.605, 0.912; *p* = 0.002) (Tables 7 and 8 and Figure 4). In a subgroup analysis within female patients (older versus younger), Cohort 1 compared to Cohort 2 was found to have increased risk of all-cause mortality (aOR 1.384, 95% CI: 1.311, 1.462; *p* < 0.001), composite outcome (aOR 1.308, 95% CI: 1.245, 1.374; *p* < 0.001), variceal bleeding (aOR 1.568, 95% CI: 1.468, 1.674; *p* < 0.001), and HCC (aOR 3.648, 95% CI: 2.481, 5.364; *p* < 0.001). There was no statistically significant difference in ascites, SBP, HRS, HPS, and HE among the two cohorts (Tables 9 and 10 and Figure 5) (Tables S4 and S5).

**Figure 6:**
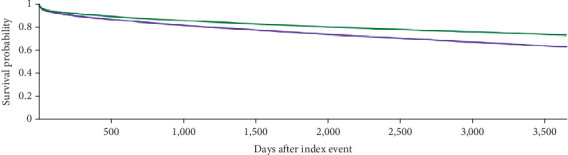
Kaplan–Meier curve (green curve = younger patients, purple curve = older patients) at 10-year interval.

**Figure 7:**
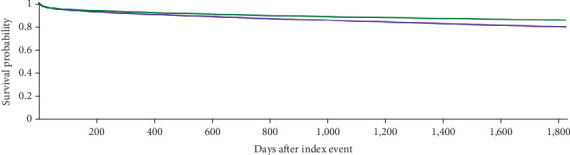
Kaplan–Meier curve (green curve = younger females, purple curve = older females) among female patients at 5-year interval.

**Figure 8:**
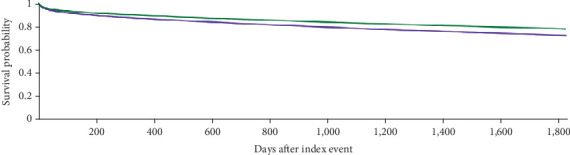
Kaplan–Meier curve (green curve = younger males, purple curve = older males) among male patients at 5-year interval.

The Kaplan–Meier survival analysis showed that 16.2% of patients in Cohort 1 and 11.9% of patients in Cohort 2 were deceased at a 10-year interval from the age of diagnosis (adjusted hazard ratio [aHR] 1.329, 95% CI: 1.293, 1.367; *p* < 0.001). Among females, 12.1% of patients in Cohort 1 and 9.0% of patients in Cohort 2 were deceased at a 5-year interval (aHR 1.307, 95% CI: 1.241, 1.376; *p* < 0.001). Among males, 15.5% of patients in Cohort 1 and 11.8% of patients in Cohort 2 were deceased at a 5-year interval (aHR 1.275, 95% CI: 1.230, 1.321; *p* < 0.001) (Table 11 and Figures 6, 7, and 8).

## 6. Discussion

This study provides a more in-depth analysis of the incidence of mortality and decompensation events in young and elderly patients with cirrhosis. It showed a significant difference in risk and survival between these age groups, highlighting the impact of aging on liver-related complications. Even though liver cirrhosis is usually more prevalent in older individuals, its occurrence in younger individuals (as early as 20) can be attributed to genetic, autoimmune liver diseases, and early acquisition of hepatitis C or B. Although our data did not have the genetic testing results in all cohorts, we acknowledge that it could have played a role specifically in the etiology of cirrhosis in younger patients.

Esophageal variceal bleeding (EVB) occurred more frequently in older patients. Research on this relationship in cirrhotic patients has yielded mixed results, with some studies indicating a greater risk in older individuals [12]. In contrast, others suggest younger patients may experience more aggressive disease progression [13, 14].

Ascites, a serious complication of cirrhosis, was more frequent in the older patients' group. The higher prevalence of EVB, HE, and ascites in the older patients' cohort in this study is most likely attributed to the fact that elderly patients have a longer duration of cirrhosis and hence a higher incidence of portal hypertension. Furthermore, the cumulative hepatic injury and age-related physiological decline may contribute to its occurrence. Older patients are at higher risk of acute renal insufficiency, regardless of the etiology of cirrhosis [15, 16]. This finding is most likely related to the fact that older patients do not tolerate diuretics well and have more risk factors for chronic renal insufficiency, including hypertension, diabetes, and sarcopenia [17].

The study found a higher incidence of SBP and HRS in younger patients. In sex-stratified analysis, the differences were statistically significant among males, whereas there was no statistically significant difference among females. The higher incidence of SBP in younger male patients may be linked to factors like increased alcohol use, contributing to faster cirrhosis progression and an increase in gut permeability for bacterial translocation [18–20]. Older patients with cirrhosis might also have a more gradual progression of liver disease, leading to a less aggressive course of SBP [21]. This interesting phenomenon warrants further investigation. The increased incidence of HRS in younger cohorts can also be explained by these findings, as clinically, SBP is a major precipitant of HRS, and as much as 30% of patients with SBP develop HRS without albumin supplementation [22]. By contrast, in younger women, estrogen's hepatoprotective effect likely blunts these responses [23]. The increased incidence of ascites, EVB, and HE in older patients highlights the need for cautious diuretic titration, routine endoscopic surveillance, and noninvasive portal pressure measurements such as platelet-based indices and liver stiffness measurement [24]. Early detection and risk stratification in these patients can guide prophylaxis with beta-blocker usage and transplant evaluation. Prioritizing SBP prophylaxis in younger patients can reduce this complication in this cohort.

On the other hand, older patients tend to have a higher prevalence of HPS compared to younger patients, though the differences were not statistically significant. The differences were insignificant in a subgroup analysis among males and females. In a study by Pascasio et al., there was no association between HPS and age, sex, or the cause of cirrhosis. Similar findings have been reported in the literature where demographic factors have not been linked as a risk factor for the development of HPS [25–28].

The older cohort displayed a significantly higher risk of developing HCC compared to the younger cohort, as indicated by the odds ratio. Existing literature indicates that age is a significant risk factor for HCC, primarily due to cumulative exposure to various etiological factors. Chronic infections with hepatitis B and C viruses are major contributors to liver carcinogenesis. Additionally, chronic alcohol consumption leading to liver cirrhosis and MASLD is also recognized as a risk factor for HCC development [29]. While younger individuals have a lower risk, their higher survival probability suggests that early detection and timely intervention may play a role in improving outcomes in this group.

Older patients have a significantly higher mortality rate compared to their younger counterparts. This finding aligns with existing literature [30]. Reduced survival in older patients may be due to declining hepatic reserves, weakened immune function, and increased susceptibility to complications like infections and multiorgan failure. These findings were persistent in sex-stratified analysis.

Comorbidities further impact prognosis, and age influences liver transplantation eligibility and outcomes. While older candidates face higher waitlist and posttransplant mortality, careful selection can still yield significant benefits [31]. The observed higher mortality in older cirrhotic patients underscores the need for tailored management strategies. This includes vigilant monitoring, early intervention for complications, and comprehensive evaluation for liver transplantation to improve outcomes in this vulnerable population.

We believe that one of the strengths of our study is a global network database and diverse patient population, along with extended follow-up periods. The differences observed were consistent at various time intervals up to 10 years from the time of diagnosis. To the best of our knowledge, this is one of the few studies that explore the differences in clinical presentation among males and females.

Our study has several limitations, including a retrospective study; reliance on ICD-10 codes; multiple centers; lack of genetic testing for younger patients; reliance on numerous physicians in estimating, managing, and diagnosing the events; and variability in recording events. Another limitation is the relatively small percentage of Asian patients in both groups, which may limit the generalizability of the study's findings to this ethnic group. Furthermore, events that occur outside the participating HCOs are not captured, and data that are documented in unstructured clinical notes are not included. These limitations stem from the nature of EHR. The mean MELD score is an estimation from the means of lab values, as TriNetX does not provide precomputed MELD values. Lastly, even after PSM, the Asian and African American races were statistically significant in both cohorts.

## 7. Conclusion

This study highlights the critical role of age at diagnosis in liver-related complications and survival outcomes in cirrhotic patients. Older individuals face a higher risk of decompensation with generally lower survival. However, certain conditions, like SBP, demonstrate unexpected trends, with younger patients experiencing higher incidences. These findings emphasize the complex interplay between aging, disease progression, and comorbidities. Given the increased vulnerability of older patients due to physiological decline and cumulative liver damage, tailored management strategies are essential. Further research is necessary to refine preventive measures, enhance early detection, and optimize treatment approaches for cirrhotic patients across different age groups.

## Figures and Tables

**Table 1:** Baseline demographics, comorbidities, and lab values before and after propensity score matching.

**Characteristic**	**Before propensity score matching**				**After propensity score matching**			
	**Older patients with liver cirrhosis (** **n** = 241, 197**)**	**Younger patients with liver cirrhosis (** **n** = 83, 024**)**	**Standardized mean difference**	**p** ** value**	**Older patients with liver cirrhosis (** **n** = 70, 983**)**	**Younger patients with liver cirrhosis (** **n** = 70, 983**)**	**Standardized mean difference**	**p** ** value**
Age, mean (SD)	69.6 (7.9)	47.9 (9.0)	2.565	< 0.001	69.1 (8.0)	47.9 (9.0)	2.493	< 0.001
Age at index	63.1 (7.5)	40.8 (7.3)	3.029	< 0.001	62.4 (7.4)	40.9 (7.3)	2.936	< 0.001
Sex								
Female	96,164 (41.3)	27,715 (38.5)	0.058	< 0.001	27,312 (38.5)	27,369 (38.6)	0.002	0.756
Male	136,715 (58.7)	44,329 (61.5)	0.058	< 0.001	43,646 (61.5)	43,583 (61.4)	0.002	0.731
Race and ethnicity								
American Indian or Alaska Native	1978 (0.8)	1615 (2.2)	0.113	< 0.001	1444 (2.0)	1441 (2.0)	< 0.001	0.955
Asian	13,253 (5.7)	6486 (9.0)	0.127	< 0.001	6661 (9.4)	6326 (8.9)	0.016	**0.002**
Black or African American	19,558 (8.4)	4956 (6.9)	0.057	< 0.001	4600 (6.5)	4909 (6.9)	0.017	**0.001**
White	147,952 (63.5)	41,612 (57.7)	0.118	< 0.001	41,413 (58.3)	41,167 (58.0)	0.007	0.186
Hispanic or Latino	21,218 (9.1)	9094 (12.6)	0.113	< 0.001	8844 (12.5)	8848 (12.5)	< 0.001	0.974
Non-Hispanic or Latino	128,744 (55.3)	38,404 (53.3)	0.040	< 0.001	37,738 (53.2)	37,777 (53.2)	0.001	0.836
Native Hawaiian or other Pacific Islander	856 (0.4)	313 (0.4)	0.011	0.011	279 (0.4)	311 (0.4)	0.007	0.187
Unknown race	40,599 (17.4)	13,269 (18.4)	0.026	< 0.001	12,991 (18.3)	13,122 (18.5)	0.005	0.37
Unknown ethnicity	83,019 (35.6)	24,577 (34.1)	0.032	< 0.001	24,401 (34.4)	24,358 (34.3)	0.001	0.81
Diagnosis								
Diabetes mellitus	40,085 (17.2)	6756 (9.4)	0.232	< 0.001	6704 (9.4)	6703 (9.4)	< 0.001	0.993
Other forms of heart disease (deprecated 2021)	24,622 (10.6)	4234 (5.9)	0.172	< 0.001	4166 (5.9)	4133 (5.8)	0.002	0.717
Ischemic heart diseases	17,674 (7.6)	1403 (1.9)	0.267	< 0.001	1349 (1.9)	1401 (2.0)	0.005	0.317
Chronic lower respiratory diseases	25,348 (10.9)	5477 (7.6)	0.113	< 0.001	5411 (7.6)	5360 (7.6)	0.003	0.609
Cerebral infarction	3856 (1.7)	327 (0.5)	0.118	< 0.001	306 (0.4)	327 (0.5)	0.004	0.403
Fatty (change of) liver, not elsewhere classified	22,491 (9.7)	7664 (10.6)	0.032	< 0.001	7318 (10.3)	7170 (10.1)	0.007	0.194
Other inflammatory liver diseases	11,705 (5.0)	3951 (5.5)	0.021	< 0.001	3627 (5.1)	3762 (5.3)	0.009	0.107
Alcoholic liver disease	5374 (2.3)	3824 (5.3)	0.157	< 0.001	2955 (4.2)	3006 (4.2)	0.004	0.5
Toxic liver disease	2103 (0.9)	856 (1.2)	0.028	< 0.001	769 (1.1)	788 (1.1)	0.003	0.628
Chronic viral hepatitis C	17,654 (7.6)	3189 (4.4)	0.133	< 0.001	3210 (4.5)	3186 (4.5)	0.002	0.759
Chronic viral hepatitis B without delta-agent^a^	3551 (1.5)	1456 (2.0)	0.038	< 0.001	1262 (1.8)	1399 (2.0)	0.014	0.007
Neoplasms	47,050 (20.2)	7020 (9.7)	0.296	< 0.001	7020 (9.9)	7003 (9.9)	0.001	0.88
Hypertensive diseases^a^	68,160 (29.3)	10,965 (15.2)	0.343	< 0.001	11,154 (15.7)	10,859 (15.3)	0.011	0.031
Other peripheral vascular diseases	5078 (2.2)	424 (0.6)	0.137	< 0.001	394 (0.6)	422 (0.6)	0.005	0.326
Autoimmune hepatitis	2151 (0.9)	957 (1.3)	0.038	< 0.001	919 (1.3)	929 (1.3)	0.001	0.815
Inflammatory liver disease, unspecified	3705 (1.6)	1498 (2.1)	0.036	< 0.001	1323 (1.9)	1366 (1.9)	0.004	0.402
Nonalcoholic steatohepatitis (NASH)	6111 (2.6)	1632 (2.3)	0.023	< 0.001	1541 (2.2)	1591 (2.2)	0.005	0.366
Alcoholic liver disease, unspecified	2573 (1.1)	1404 (1.9)	0.069	< 0.001	1193 (1.7)	1242 (1.7)	0.005	0.317
Alcoholic hepatitis	2285 (1.0)	2409 (3.3)	0.163	< 0.001	1648 (2.3)	1659 (2.3)	0.001	0.847
Alcoholic fatty liver	933 (0.4)	486 (0.7)	0.037	< 0.001	386 (0.5)	413 (0.6)	0.005	0.338
Laboratory values, mean (SD)								
Bilirubin total (mass/volume) in serum, plasma, or blood	1.2 (2.6)	1.8 (3.9)	0.186	< 0.001	1.4 (2.8)	1.8 (3.8)	0.001	0.828
INR in plasma or blood	1.2 (0.4)	1.2 (0.4)	0.048	< 0.001	1.2 (0.4)	1.2 (0.4)	0.008	0.146
Creatinine (mass/volume) in serum, plasma, or blood	1.1 (4.2)	0.9 (3.0)	0.043	< 0.001	1.1 (4.3)	0.9 (3.0)	0.001	0.863
Sodium (moles/volume) in serum, plasma, or blood	138.2 (4.0)	138.0 (3.7)	0.061	< 0.001	138.2 (4.0)	138.0 (3.7)	0.001	0.816
Albumin (mass/volume) in serum, plasma, or blood	3.8 (0.7)	3.8 (0.8)	0.068	< 0.001	3.7 (0.7)	3.8 (0.7)	< 0.001	0.987
BMI	30.0 (7.3)	30.8 (8.9)	0.095	< 0.001	29.5 (7.2)	30.9 (8.9)	0.001	0.822

*Note:* Bolded data are outcomes that were statistically significant after PSM.

^a^Outcomes were statistically significant even after PSM.

**Table 2:** Progression of MELD 3.0.

**Cohort**	**Sex**	**MELD 3.0 at diagnosis**	**MELD 3.0 at 5-year interval**	**MELD 3.0 at 10-year interval**
Cohort 1	Male	10	17	20
Cohort 1	Female	12	18	21
Cohort 2	Male	10	17	19
Cohort 2	Female	12	18	20

**Table 3:** Lab test values in patients with liver cirrhosis at 5 years. INR, international normalized ratio.

**Lab test**	**M** **e** **a** **n** + **SD of Cohort 1**	**M** **e** **a** **n** + **SD of Cohort 2**	**p** ** value**
Aspartate aminotransferase (enzymatic activity/volume)	92.257 ± 526.859	109.116 ± 565.823	< 0.001
Alanine aminotransferase (enzymatic activity/volume)	51.636 ± 167.841	61.471 ± 174.168	< 0.001
INR in plasma or blood	1.333 ± 0.700	1.416 ± 0.834	< 0.001
Alkaline phosphatase (enzymatic activity/volume)	126.104 ± 119.816	130.993 ± 124.333	< 0.001
Albumin (mass/volume) in serum, plasma, or blood	3.545 ± 0.840	3.621 ± 0.865	0.337
Bilirubin (mass/volume) in serum, plasma, or blood	2.324 ± 5.516	3.403 ± 7.273	0.008
Creatinine (mass/volume) in serum, plasma, or blood	1.455 ± 6.787	1.212 ± 4.718	0.001
Serum sodium (moles/volume) in serum, plasma, or blood	138.132 ± 4.865	137.839 ± 4.455	< 0.001

**Table 4:** Lab test values in patients with liver cirrhosis at 10 years. INR, international normalized ratio.

**Lab test**	**M** **e** **a** **n** ± **SD of Cohort 1**	**M** **e** **a** **n** ± **SD of Cohort 2**	**p** ** value**
Aspartate aminotransferase (enzymatic activity/volume)	94.412 ± 526.389	108.972 ± 569.335	< 0.001
Alanine aminotransferase (enzymatic activity/volume)	51.19 ± 167.438	60.873 ± 175.947	< 0.001
INR in plasma or blood	1.330 ± 0.690	1.411 ± 0.829	< 0.001
Alkaline phosphatase (enzymatic activity/volume)	126.975 ± 121.350	130.609 ± 124.077	< 0.001
Albumin (mass/volume) in serum, plasma, or blood	3.538 ± 0.841	3.627 ± 0.865	0.177
Bilirubin (mass/volume) in serum, plasma, or blood	2.385 ± 5.829	3.388 ± 7.315	0.001
Creatinine (mass/volume) in serum, plasma, or blood	1.606 ± 7.941	1.29 ± 5.489	0.008
Serum sodium (moles/volume) in serum, plasma, or blood	138.073 ± 5.06	137.846 ± 4.538	< 0.001

**Table 5:** Outcomes in patients with liver cirrhosis at 5 years.

**Outcome**	**Cohort**	**Patients in cohort**	**Patients with outcome**	**Risk**	**Odds ratio**	**95% CI**	**p** ** value**
Variceal bleeding	1	70,983	6086	0.086	1.309	(1.258, 1.361)	< 0.001
	2	70,983	4747	0.067			
Ascites	1	70,983	2554	0.036	1.114	(1.052, 1.180)	< 0.001
	2	70,983	2301	0.032			
SBP	1	70,983	268	0.004	0.848	(0.720, 0.998)	0.023
	2	70,983	316	0.004			
HRS	1	70,983	322	0.005	0.753	(0.651, 0.871)	< 0.001
	2	70,983	427	0.006			
Mortality	1	70,983	10,170	0.143	1.378	(1.335, 1.422)	< 0.001
	2	70,983	7682	0.108			
HPS	1	70,983	29	0.000	1.45	(0.820, 2.564)	0.101
	2	70,983	20	0.000			
Hepatic encephalopathy	1	70,983	1682	0.024	1.1	(1.026, 1.180)	0.004
	2	70,983	1532	0.022			
Rates of hospitalization	1	70,983	25,804	0.364	1.009	(0.987, 1.031)	0.218
	2	70,983	25,663	0.362			
HCC	1	70,983	547	0.008	2.924	(2.477, 3.453)	< 0.001
	2	70,983	188	0.003			
Composite outcome	1	70,983	10,537	0.148	1.236	(1.199, 1.275)	< 0.001
	2	70,983	8772	0.124			

**Table 6:** Outcomes in patients with liver cirrhosis at 10 years.

**Outcome**	**Cohort**	**Patients in cohort**	**Patients with outcome**	**Risk**	**Odds ratio**	**95% CI**	**p** ** value**
Variceal bleeding	1	70,983	6482	0.091	1.337	(1.287, 1.390)	< 0.001
	2	70,983	4962	0.070			
Ascites	1	70,983	2721	0.038	1.134	(1.073, 1.199)	< 0.001
	2	70,983	2410	0.034			
SBP	1	70,983	283	0.004	0.881	(0.751, 1.034)	0.061
	2	70,983	321	0.005			
HRS	1	70,983	337	0.005	0.77	(0.688, 0.888)	< 0.001
	2	70,983	437	0.006			
Mortality	1	70,983	11,480	0.162	1.418	(1.376, 1.462)	< 0.001
	2	70,983	8501	0.120			
HPS	1	70,983	30	0.000	1.429	(0.818, 2.496)	0.105
	2	70,983	21	0.000			
Hepatic encephalopathy	1	70,983	1771	0.025	1.128	(1.053, 1.208)	< 0.001
	2	70,983	1575	0.022			
Rates of hospitalization	1	70,983	27,087	0.382	1.014	(0.992, 1.036)	0.104
	2	70,983	26,857	0.378			
HCC	1	70,983	588	0.008	3.032	(2.578, 3.567)	< 0.001
	2	70,983	195	0.003			
Composite outcome	1	70,983	11,147	0.157	1.266	(1.229, 1.305)	< 0.001
	2	70,983	9103	0.128			

**Table 7:** Outcomes in male patients with liver cirrhosis at 5 years.

**Outcome**	**Cohort**	**Patients in cohort**	**Patients with outcome**	**Risk**	**Odds ratio**	**95% CI**	**p** ** value**
Variceal bleeding	1	44,315	3875	0.087	1.208	(1.150, 1.268)	< 0.001
	2	44,315	3258	0.074			
Ascites	1	44,315	1633	0.037	1.207	(1.122, 1.299)	< 0.001
	2	44,315	1361	0.031			
SBP	1	44,315	160	0.004	0.743	(0.605, 0.912)	0.002
	2	44,315	215	0.005			
HRS	1	44,315	241	0.005	0.827	(0.697, 0.982)	0.015
	2	44,315	291	0.007			
Mortality	1	44,315	6890	0.155	1.37	(1.319, 1.424)	< 0.001
	2	44,315	5248	0.118			
HPS	1	44,315	14	0.000	1.273	(0.578, 2.804)	0.275
	2	44,315	11	0.000			
Hepatic encephalopathy	1	44,315	1070	0.024	1.19	(1.088, 1.301)	< 0.001
	2	44,315	903	0.020			
Rates of hospitalization	1	44,315	16,308	0.368	1.037	(1.009, 1.066)	0.005
	2	44,315	15,935	0.360			
HCC	1	44,315	434	0.010	2.782	(2.317, 3.340)	< 0.001
	2	44,315	157	0.004			
Composite outcome	1	44,315	6762	0.153	1.216	(1.171, 1.264)	< 0.001
	2	44,315	5714	0.129			

**Table 8:** Lab test values for male patients with liver cirrhosis at 5 years. INR, international normalized ratio.

**Lab test**	**M** **e** **a** **n** + **SD of Cohort 1**	**M** **e** **a** **n** + **SD of Cohort 2**	**p** ** value**
Aspartate aminotransferase (enzymatic activity/volume)	98.389 ± 540.782	120.612 ± 608.004	< 0.001
Alanine aminotransferase (enzymatic activity/volume)	55.688 ± 175.238	68.994 ± 193.758	< 0.001
INR in plasma or blood	1.354 ± 0.693	1.433 ± 0.838	0.001
Alkaline phosphatase (enzymatic activity/volume)	127.836 ± 123.196	133.058 ± 128.260	< 0.001
Albumin (mass/volume) in serum, plasma, or blood	3.511 ± 0.853	3.612 ± 0.890	0.436
Bilirubin (mass/volume) in serum, plasma, or blood	2.613 ± 6.022	3.649 ± 7.496	0.010
Creatinine (mass/volume) in serum, plasma, or blood	1.608 ± 7.481	1.247 ± 4.355	0.009
Serum sodium (moles/volume) in serum, plasma, or blood	137.772 ± 5.098	137.716 ± 4.643	0.209

**Table 9:** Outcomes in female patients with liver cirrhosis at 5 years.

**Outcome**	**Cohort**	**Patients in cohort**	**Patients with outcome**	**Risk**	**Odds ratio**	**95% CI**	**p** ** value**
Variceal bleeding	1	27,902	2409	0.086	1.568	(1.468, 1.674)	< 0.001
	2	27,902	1586	0.057			
Ascites	1	27,902	1022	0.037	1.027	(0.940, 1.123)	0.278
	2	27,902	996	0.036			
SBP	1	27,902	77	0.003	0.754	(0.561, 1.014)	0.031
	2	27,902	102	0.004			
HRS	1	27,902	120	0.004	0.844	(0.662, 1.077)	0.087
	2	27,902	142	0.005			
Mortality	1	27,902	3376	0.121	1.384	(1.311, 1.462)	< 0.001
	2	27,902	2524	0.090			
HPS	1	27,902	15	0.001	1.5	(0.674, 3.340)	0.16
	2	27,902	10	0.000			
Hepatic encephalopathy	1	27,902	689	0.025	1.052	(0.944, 1.172)	0.181
	2	27,902	656	0.024			
Rates of hospitalization	1	27,902	9856	0.353	0.942	(0.910, 0.975)	< 0.001
	2	27,902	10,240	0.367			
HCC	1	27,902	120	0.004	3.648	(2.481, 5.364)	< 0.001
	2	27,902	33	0.001			
Composite outcome	1	27,902	4087	0.146	1.308	(1.245, 1.374)	< 0.001
	2	27,902	3236	0.116			

**Table 10:** Lab test values in female patients with liver cirrhosis at 5 years. INR, international normalized ratio.

**Lab test**	**M** **e** **a** **n** + **SD of Cohort 1**	**M** **e** **a** **n** + **SD of Cohort 2**	**p** ** value**
Aspartate aminotransferase (enzymatic activity/volume)	78.185 ± 427.516	89.753 ± 446.289	0.797
Alanine aminotransferase (enzymatic activity/volume)	43.48 ± 127.343	50.009 ± 137.87	0.006
INR in plasma or blood	1.302 ± 0.733	1.384 ± 0.828	< 0.001
Alkaline phosphatase (enzymatic activity/volume)	126.447 ± 115.729	129.340 ± 126.060	0.004
Albumin (mass/volume) in serum, plasma, or blood	3.611 ± 0.808	3.637 ± 0.825	0.348
Bilirubin (mass/volume) in serum, plasma, or blood	1.888 ± 4.520	2.942 ± 6.690	0.04
Creatinine (mass/volume) in serum, plasma, or blood	1.166 ± 5.158	1.123 ± 4.986	0.003
Serum sodium (moles/volume) in serum, plasma, or blood	138.639 ± 4.471	138.012 ± 4.148	< 0.001

**Table 11:** Survival analysis table.

**Cohort**	**Patients in cohort**	**Patients with outcome**	**Adjusted hazard ratio (aHR)**	**p**	**95% CI**
Survival analysis at 10-year interval among patients with liver cirrhosis					
Cohort 1	70,983	11,480	1.329	< 0.001	1.293, 1.367
Cohort 2	70,983	8501			
Survival analysis at 5-year interval among male patients with liver cirrhosis					
Cohort 1	44,315	6890	1.275	< 0.001	1.230, 1.321
Cohort 2	44,315	5248			
Survival analysis at 5-year interval among female patients with liver cirrhosis					
Cohort 1	27,902	3376	1.307	< 0.0001	1.241, 1.376
Cohort 2	27,902	2524			

## Data Availability

The data that support the findings of this study are available from TriNetX, LLC, but third-party restrictions apply to the availability of these data. The data were used under license for this study, with restrictions that do not allow for the data to be redistributed or made publicly available. However, for accredited researchers, the TriNetX data is available for licensing at TriNetX, LLC. Data access may require a data sharing agreement and may incur data access fees.

## Supporting Information

Supporting Information Additional supporting information can be found online in the Supporting Information section. Table S1 lists the inclusion and exclusion criteria. Table S2 summarizes the ICD, CPT, and SNOMED codes used to define study outcomes. Table S3 includes TriNetX curated laboratory codes for various tests. Tables S4 and S5 include baseline demographics, comorbidities, and laboratory values before and after PSM for male and female subgroups (Tables S1, S2, S3, S4, and S5).

## References

[1] Duo H., You J., Du S. (2025). Liver Cirrhosis in 2021: Global Burden of Disease Study. *PLoS One*.

[2] Tham E. K. J., Tan D. J. H., Danpanichkul P. (2025). The Global Burden of Cirrhosis and Other Chronic Liver Diseases in 2021. *Liver International*.

[3] Huang D. Q., Terrault N. A., Tacke F. (2023). Global Epidemiology of Cirrhosis — Aetiology, Trends and Predictions. *Nature Reviews Gastroenterology & Hepatology*.

[4] Le P., Tatar M., Dasarathy S. (2025). Estimated Burden of Metabolic Dysfunction–Associated Steatotic Liver Disease in US Adults, 2020 to 2050. *JAMA Network Open*.

[5] Hunt N. J., Kang S. W., Lockwood G. P., Le Couteur D. G., Cogger V. C. (2019). Hallmarks of Aging in the Liver. *Computational and Structural Biotechnology Journal*.

[6] Wynne H. A., Cope L. H., Mutch E., Rawlins M. D., Woodhouse K. W., James O. F. W. (1989). The Effect of Age Upon Liver Volume and Apparent Liver Blood Flow in Healthy Man. *Hepatology*.

[7] Al-Smadi K., Qureshi A., Buitrago M., Ashouri B., Kayali Z. (2024). Survival and Disease Progression in Older Adult Patients With Cirrhosis: A Retrospective Study. *International Journal of Hepatology*.

[8] Zhou J., Huang Z., Chen Z., Xu F., Tong R., Zheng S. (2021). Impact of Donor Age on Liver Transplant Outcomes in Patients With Hepatocellular Carcinoma: Analysis of the SRTR Database. *BMC Gastroenterology*.

[9] Schmucker D. L., Sanchez H. (2011). Liver Regeneration and Aging: A Current Perspective. *Current Gerontology and Geriatrics Research*.

[10] Ronco C., Bellomo R., McCullough P. A. (2010). *Cardiorenal Syndromes in Critical Care, Contributions to Nephrology*.

[11] Ligthart-Melis G. C., Luiking Y. C., Kakourou A., Cederholm T., Maier A. B., De Van Der Schueren M. A. E. (2020). Frailty, Sarcopenia, and Malnutrition Frequently (Co-)occur in Hospitalized Older Adults: A Systematic Review and Meta-analysis. *Journal of the American Medical Directors Association*.

[12] Garzon M., Bernal M. L., Ramirez D. (2023). P-124 Title: Clinical Characteristics of Cirrhotic Patients With Variceal Bleeding in a Single Center EXPERIENCE. Descriptive Study. *Annals of Hepatology*.

[13] Kim Y. D., Cheon G. J., Kim M. Y., Suk K. T., Baik S. K., Kim D. J. (2012). Changes in the Clinical Outcomes of Variceal Bleeding in Cirrhotic Patients: A 10-Year Experience in Gangwon Province, South Korea. *Gut Liver*.

[14] Vara-Luiz F., Mendes I., Palma C. (2025). Upper Gastrointestinal Bleeding Differences Between Older and Younger Adults: Should Bleeding in Non-Cirrhotic Patients Be Considered a Geriatric Syndrome?. *Therapeutic Advances in Gastroenterology*.

[15] Carrier P., Loustaud-Ratti V., Debette-Gratien M., Elkrief L. (2024). Ascites in Cirrhotic Patients: A Comprehensive Review. *Exploration of Digestive Diseases*.

[16] Fernández-Esparrach G., Sánchez-Fueyo A., Ginès P. (2001). A Prognostic Model for Predicting Survival in Cirrhosis With Ascites. *Journal of Hepatology*.

[17] Terbah R., Testro A., Gow P., Majumdar A., Sinclair M. (2023). Portal Hypertension in Malnutrition and Sarcopenia in Decompensated Cirrhosis-Pathogenesis, Implications and Therapeutic Opportunities. *Nutrients*.

[18] Wiest R., Lawson M., Geuking M. (2014). Pathological Bacterial Translocation in Liver Cirrhosis. *Journal of Hepatology*.

[19] Skinner C., Thompson A. J., Thursz M. R., Marchesi J. R., Vergis N. (2020). Intestinal Permeability and Bacterial Translocation in Patients With Liver Disease, Focusing on Alcoholic Aetiology: Methods of Assessment and Therapeutic Intervention. *Therapeutic Advances in Gastroenterology*.

[20] Hartmann P., Chen W.-C., Schnabl B. (2012). The Intestinal Microbiome and the Leaky Gut as Therapeutic Targets in Alcoholic Liver Disease. *Frontiers in physiology,*.

[21] European Association For The Study Of The Liver (2010). EASL Clinical Practice Guidelines on the Management of Ascites, Spontaneous Bacterial Peritonitis, and Hepatorenal Syndrome in cirrhosis. *Journal of Hepatology*.

[22] Angeli P., Bernardi M., Villanueva C. (2018). EASL Clinical Practice Guidelines for the Management of Patients With Decompensated Cirrhosis. *Journal of Hepatology*.

[23] Xu L., Yuan Y., Che Z. (2022). The Hepatoprotective and Hepatotoxic Roles of Sex and Sex-Related Hormones. *Frontiers in Immunology*.

[24] De Franchis R. (2015). Expanding Consensus in Portal Hypertension. *Journal of Hepatology*.

[25] Ferreira P. P., Camara E. J. N., Paula R. L. P. D., Zollinger C. C., Cavalcanti A. R., Bittencourt P. L. (2008). Prevalence of Hepatopulmonary Syndrome in Patients With Decompensated Chronic Liver Disease and Its Impact on Short-Term Survival. *Arquivos de Gastroenterologia*.

[26] Pascasio J. M., Grilo I., López-Pardo F. J. (2014). Prevalence and Severity of Hepatopulmonary Syndrome and Its Influence on Survival in Cirrhotic Patients Evaluated for Liver Transplantation. *American Journal of Transplantation*.

[27] Varghese J., Ilias-basha H., Dhanasekara R., Singh S., Venkataraman J. (2007). Hepatopulmonary Syndrome - Past to Present. *Annals of Hepatology*.

[28] Younis I., Sarwar S., Butt Z., Tanveer S., Qaadir A., Jadoon N. A. (2015). Clinical Characteristics, Predictors, and Survival Among Patients With Hepatopulmonary Syndrome. *Annals of Hepatology*.

[29] Haydon G. H., Hayes P. C. (1995). Hepatocellular Carcinoma. *British Journal of Hospital Medicine*.

[30] Abu-Freha N., Estis-Deaton A., Aasla M. (2022). Liver Cirrhosis in Elderly Patients: Clinical Characteristics, Complications, and Survival—Analyses From a Large Retrospective Study. *Aging Clinical and Experimental Research*.

[31] Durand F., Levitsky J., Cauchy F., Gilgenkrantz H., Soubrane O., Francoz C. (2019). Age and Liver Transplantation. *Journal of Hepatology*.

