# Supplementary material for: Impact of Age on Mortality and Decompensation Events in Patients With Liver Cirrhosis: A Multicenter, Propensity Score Matched Study
Source: Int J Hepatol. 2025 Nov 24;2025:8852224. doi: 10.1155/ijh/8852224 (PMC12668842; doi:10.1155/ijh/8852224)
Supplement: Supporting Information — Additional supporting information can be found online in the Supporting Information section. Table S1 lists the inclusion and exclusion criteria. Table S2 summarizes the ICD, CPT, and SNOMED codes used to define study outcomes. Table S3 includes TriNetX curated laboratory codes for various tests. Tables S4 and S5 include baseline demographics, comorbidities, and laboratory values before and after PSM for male and female subgroups (Tables S1, S2, S3, S4, and S5). [file 8852224.f1.docx]

**Supplementary Table 1(Inclusion and Exclusion Criteria)**

| **Outcome** | **ICD Code** |
| --- | --- |
| Liver Cirrhosis (between 51 and 80 years old at event) | ICD10CM:K70.3, K74.69, K74.60, K74.4, K74.5, K74.6 |
| Liver Cirrhosis (between 20 and 50 years old at event) | ICD10CM:K70.3, K74.69, K74.60, K74.4, K74.5, K74.6 |
| **EXCLUSION CRITERIA** |  |
| Human immunodeficiency virus [HIV] disease | ICD10CM:B20 |
| Heart failure | ICD10CM:I50 |
| End stage renal disease | ICD10CM:N18.6 |
| Malignant (primary) neoplasm, unspecified | ICD10CM:C80.1 |
| Other psychoactive substance dependence, uncomplicated | ICD10CM:F19.20 |
| Chronic kidney disease, stage 4 (severe) | ICD10CM:N18.4 |
| Chronic kidney disease, stage 5 | ICD10CM:N18.5 |
| Other psychoactive substance abuse | ICD10CM:F19.1 |
| Other stimulant abuse, uncomplicated | ICD10CM:F15.10 |
| Other stimulant dependence, uncomplicated | ICD10CM:F15.20 |
| Other stimulant use, unspecified with stimulant-induced psychotic disorder, unspecified | ICD10CM:F15.959 |
| Other stimulant use, unspecified with stimulant-induced anxiety disorder | ICD10CM:F15.980 |
| Other stimulant use, unspecified with intoxication, unspecified | ICD10CM:F15.929 |
| **Excluded if any incidence prior to the onset of liver cirrhosis** |  |
| Hepatic encephalopathy | ICD10CM:K76.82 |
| Alcoholic hepatic failure with coma | ICD10CM:K70.41 |
| Chronic hepatic failure with coma | ICD10CM:K72.11 |
| Malignant neoplasm of liver, primary, unspecified as to type | ICD10CM:C22.8 |
| Malignant neoplasm of liver, not specified as primary or secondary | ICD10CM:C22.9 |
| Hepatopulmonary syndrome | ICD10CM:K76.81 |
| Ascites | ICD10CM:R18 |
| Alcoholic hepatitis with ascites | ICD10CM:K70.11 |
| Alcoholic cirrhosis of liver with ascites | ICD10CM:K70.31 |
| Esophageal varices with bleeding | ICD10CM:I85.01 |
| Secondary esophageal varices with bleeding | ICD10CM:I85.11 |
| Hepatorenal syndrome | ICD10CM:K76.7 |

**Supplementary Table 2 (List of ICD Codes for Outcomes)**

| **Outcome** | **ICD Code** |
| --- | --- |
| Variceal Bleeding | ICD10CM:I85.11; ICD10CM:I85.01 |
| Ascites | ICD10CM:R18 |
| SBP | ICD10CM:K65.2 |
| HRS | ICD10CM:K76.7 |
| Mortality | Deceased |
| HPS | ICD10CM:K76.81 |
| Hepatic Encephalopathy | ICD10CM:K76.82; ICD10CM:K70.41; ICD10CM:K72.11; ICD10CM:K72.91; ICD10CM:B15.0; ICD10CM:B16.0; ICD10CM:B16.2; ICD10CM:B17.11; ICD10CM:B19.0; ICD10CM:B19.11; ICD10CM:B19.21 |
| Rates of Hospitalization | HL7V3.0: Visit Type: IMP; HL7V3.0: Visit Type:ACUTE; HL7V3.0: Visit Type: NONAC; HL7V3.0: Visit Type:SS; CPT:1013659; CPT:1013699; CPT:1013729; SNOMED:53923005; SNOMED:24882007; SNOMED:394656005; SNOMED:737481003; SNOMED:86181006 |
| HCC | ICD10CM:C22.8, C22.9 |
| Composite Outcome | ICD10CM:K65.2; ICD10CM:K76.81; ICD10CM:K76.7; ICD10CM:R18; ICD10CM:C22.8; ICD10CM:C22.9; ICD10CM:K76.82; ICD10CM:I85.11; ICD10CM:I85.01; ICD10CM:K70.41; ICD10CM:K72.11; ICD10CM:K72.91; ICD10CM:B15.0; ICD10CM:B16.0; ICD10CM:B16.2; ICD10CM:B17.11; ICD10CM:B19.21; ICD10CM:B19.11; ICD10CM:B19.0 |

**Supplementary Table 3: TNX** Codes for Lab Tests in Patients with Liver Cirrhosis

| **Outcome** | **TNX Code** |
| --- | --- |
| Aspartate aminotransferase [Enzymatic activity/volume] in Serum or Plasma | TNX 9047 |
| Alanine aminotransferase [Enzymatic activity/volume] in Serum, Plasma or Blood | TNX 9044 |
| INR in Plasma or Blood | TNX 9032 |
| Alkaline phosphatase [Enzymatic activity/volume] in Serum, Plasma or Blood | TNX 9046 |
| Albumin [Mass/volume] in Serum, Plasma or Blood | TNX 9045 |
| Bilirubin total [Mass/volume] in Serum, Plasma or Blood | TNX 9050 |
| Creatinine [Mass/volume] in Serum, Plasma or Blood | TNX 9024 |
| Sodium [Moles/volume] in Serum, Plasma or Blood | TNX 9029 |

**Supplementary Table 4 (Baseline Characteristics of Male Patients with Liver Cirrhosis Before and After Propensity Score Matching)**

| **Cohort 1 and cohort 2 patient count before and after propensity score matching** | | | | | | | | | | | | | | |
| --- | --- | --- | --- | --- | --- | --- | --- | --- | --- | --- | --- | --- | --- | --- |
|  | | | Cohort | | | Patient count before matching | | | | | Patient count after matching | | | |
|  | | | 1 - 51 to 80 males only | | | 143,664 | | | | | 44,315 | | | |
|  | | | 2 - 20 to 50 males only | | | 51,866 | | | | | 44,315 | | | |
| **Propensity score density function - Before and after matching (cohort 1 - purple, cohort 2 - green)** | | | | | | | | | | | | | | |
|  |  | | 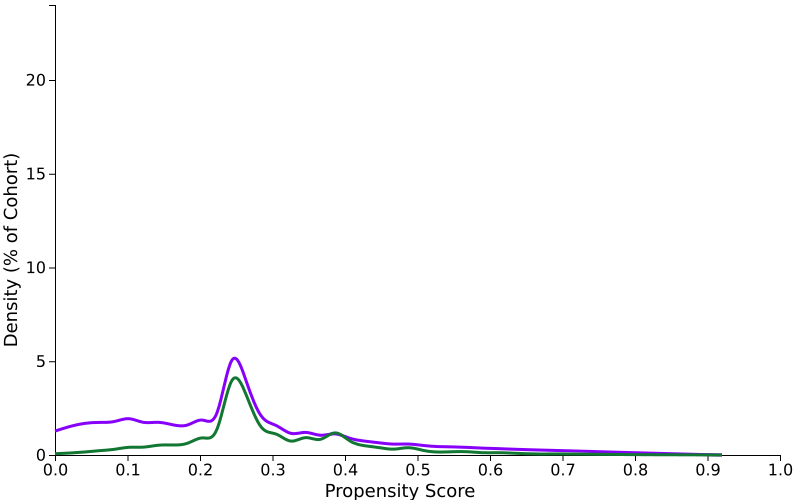 | | | | 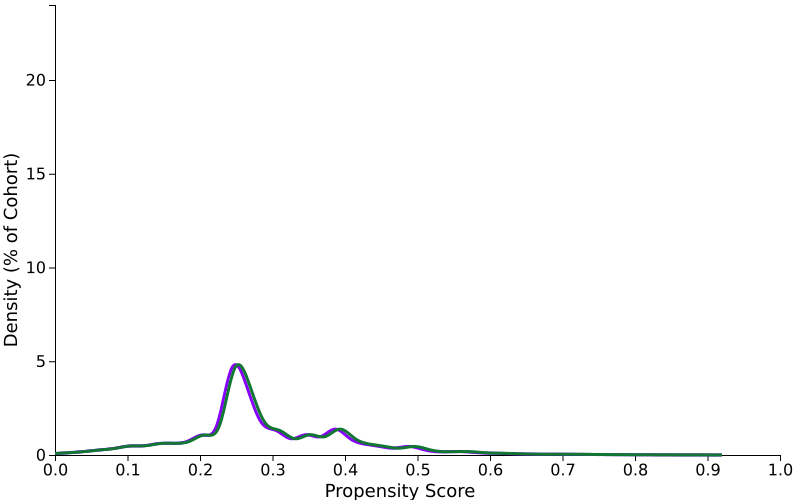 | | | | | | | |
| **Cohort 1 (N = 143,664) and cohort 2 (N = 51,866) characteristics before propensity score matching** | | | | | | | | | | | | | | |
|  | **Demographics** | | | | | | | | | | | | | |
|  |  | Cohort | | |  | Mean ± SD | | Patients | | % of Cohort | | | P-Value | Std diff. |
|  |  | 1 2 | | Age | Current Age | 69.2 +/- 7.8 48.2 +/- 8.8 | | 138,875 45,038 | | 100% 100% | | | <0.001 | 2.522 |
|  |  | 1 2 | | AI | Age at Index | 62.7 +/- 7.3 41.1 +/- 7.1 | | 138,875 45,038 | | 100% 100% | | | <0.001 | 3.001 |
|  |  | 1 2 | | F | Female |  | | 0 0 | | 0% 0% | | | -- | -- |
|  |  | 1 2 | | 2054-5 | Black or African American |  | | 11,963 2,687 | | 8.6% 6.0% | | | <0.001 | 0.102 |
|  |  | 1 2 | | M | Male |  | | 138,875 45,038 | | 100% 100% | | | -- | -- |
|  |  | 1 2 | | 2106-3 | White |  | | 85,323 24,698 | | 61.4% 54.8% | | | <0.001 | 0.134 |
|  |  | 1 2 | | 1002-5 | American Indian or Alaska Native |  | | 1,058 969 | | 0.8% 2.2% | | | <0.001 | 0.116 |
|  |  | 1 2 | | UNK | Unknown Race |  | | 26,557 9,295 | | 19.1% 20.6% | | | <0.001 | 0.038 |
|  |  | 1 2 | | 2076-8 | Native Hawaiian or Other Pacific Islander |  | | 521 206 | | 0.4% 0.5% | | | 0.016 | 0.013 |
|  |  | 1 2 | | UN | Unknown Ethnicity |  | | 51,316 16,025 | | 37.0% 35.6% | | | <0.001 | 0.029 |
|  |  | 1 2 | | 2135-2 | Hispanic or Latino |  | | 11,930 6,140 | | 8.6% 13.6% | | | <0.001 | 0.161 |
|  |  | 1 2 | | 2186-5 | Not Hispanic or Latino |  | | 75,629 22,873 | | 54.5% 50.8% | | | <0.001 | 0.074 |
|  |  | 1 2 | | 2028-9 | Asian |  | | 8,022 4,476 | | 5.8% 9.9% | | | <0.001 | 0.155 |
|  | **Diagnosis** | | | | | | | | | | | | | |
|  |  | Cohort | | |  | Mean ± SD | | Patients | | % of Cohort | | | P-Value | Std diff. |
|  |  | 1 2 | | E08-E13 | Diabetes mellitus |  | | 20,817 3,647 | | 15.0% 8.1% | | | <0.001 | 0.217 |
|  |  | 1 2 | | I30-I52 | Other forms of heart disease (deprecated 2021) |  | | 14,053 2,341 | | 10.1% 5.2% | | | <0.001 | 0.186 |
|  |  | 1 2 | | I20-I25 | Ischemic heart diseases |  | | 11,055 855 | | 8.0% 1.9% | | | <0.001 | 0.283 |
|  |  | 1 2 | | J40-J4A | Chronic lower respiratory diseases |  | | 12,373 2,386 | | 8.9% 5.3% | | | <0.001 | 0.141 |
|  |  | 1 2 | | I63 | Cerebral infarction |  | | 2,302 176 | | 1.7% 0.4% | | | <0.001 | 0.126 |
|  |  | 1 2 | | K76.0 | Fatty (change of) liver, not elsewhere classified |  | | 10,463 4,006 | | 7.5% 8.9% | | | <0.001 | 0.050 |
|  |  | 1 2 | | K75 | Other inflammatory liver diseases |  | | 4,840 1,870 | | 3.5% 4.2% | | | <0.001 | 0.035 |
|  |  | 1 2 | | K70 | Alcoholic liver disease |  | | 3,738 2,524 | | 2.7% 5.6% | | | <0.001 | 0.146 |
|  |  | 1 2 | | K71 | Toxic liver disease |  | | 1,065 464 | | 0.8% 1.0% | | | <0.001 | 0.028 |
|  |  | 1 2 | | B18.2 | Chronic viral hepatitis C |  | | 11,706 2,094 | | 8.4% 4.6% | | | <0.001 | 0.153 |
|  |  | 1 2 | | B18.1 | Chronic viral hepatitis B without delta-agent |  | | 2,427 1,005 | | 1.7% 2.2% | | | <0.001 | 0.035 |
|  |  | 1 2 | | C00-D49 | Neoplasms |  | | 25,957 3,215 | | 18.7% 7.1% | | | <0.001 | 0.350 |
|  |  | 1 2 | | I10-I1A | Hypertensive diseases |  | | 37,923 6,652 | | 27.3% 14.8% | | | <0.001 | 0.311 |
|  |  | 1 2 | | I73 | Other peripheral vascular diseases |  | | 2,929 161 | | 2.1% 0.4% | | | <0.001 | 0.159 |
|  |  | 1 2 | | K75.4 | Autoimmune hepatitis |  | | 431 306 | | 0.3% 0.7% | | | <0.001 | 0.053 |
|  |  | 1 2 | | K75.9 | Inflammatory liver disease, unspecified |  | | 1,966 824 | | 1.4% 1.8% | | | <0.001 | 0.033 |
|  |  | 1 2 | | K75.81 | Nonalcoholic steatohepatitis (NASH) |  | | 2,326 728 | | 1.7% 1.6% | | | 0.399 | 0.005 |
|  |  | 1 2 | | K70.9 | Alcoholic liver disease, unspecified |  | | 1,782 950 | | 1.3% 2.1% | | | <0.001 | 0.064 |
|  |  | 1 2 | | K70.1 | Alcoholic hepatitis |  | | 1,585 1,538 | | 1.1% 3.4% | | | <0.001 | 0.153 |
|  |  | 1 2 | | K70.0 | Alcoholic fatty liver |  | | 628 319 | | 0.5% 0.7% | | | <0.001 | 0.034 |
|  | **Laboratory** | | | | | | | | | | | | | |
|  |  | Cohort | | |  | Mean ± SD | | P-Value | Std diff. | | |  |  |  |
|  |  | 1 2 | | 9050 | Bilirubin.total [Mass/volume] in Serum, Plasma or Blood | 1.3 +/- 2.8 2.0 +/- 4.0 | | <0.001 | 0.188 | | |  |  |  |
|  |  | 1 2 | |  | 0 - 0 mg/dL |  | | <0.001 | 0.033 | | |  |  |  |
|  |  | 1 2 | | 9032 | INR in Plasma or Blood | 1.2 +/- 0.4 1.2 +/- 0.4 | | <0.001 | 0.046 | | |  |  |  |
|  |  | 1 2 | |  | 0 - 0 {INR} |  | | <0.001 | 0.065 | | |  |  |  |
|  |  | 1 2 | | 9024 | Creatinine [Mass/volume] in Serum, Plasma or Blood | 1.2 +/- 4.8 1.0 +/- 2.8 | | <0.001 | 0.054 | | |  |  |  |
|  |  | 1 2 | |  | 0 - 0 mg/dL |  | | <0.001 | 0.054 | | |  |  |  |
|  |  | 1 2 | | 9029 | Sodium [Moles/volume] in Serum, Plasma or Blood | 137.9 +/- 4.1 138.0 +/- 3.9 | | 0.077 | 0.016 | | |  |  |  |
|  |  | 1 2 | |  | 0 - 0 mmol/L |  | | <0.001 | 0.062 | | |  |  |  |
|  |  | 1 2 | | 9045 | Albumin [Mass/volume] in Serum, Plasma or Blood | 3.8 +/- 0.7 3.9 +/- 0.8 | | <0.001 | 0.135 | | |  |  |  |
|  |  | 1 2 | |  | 0 - 0 g/dL |  | | <0.001 | 0.043 | | |  |  |  |
|  |  | 1 2 | | 9083 | BMI | 29.2 +/- 6.5 30.0 +/- 7.9 | | <0.001 | 0.099 | | |  |  |  |
|  |  | 1 2 | |  | 0 - 0 kg/m2 |  | | <0.001 | 0.070 | | |  |  |  |
| **Cohort 1 (N = 44,315) and cohort 2 (N = 44,315) characteristics after propensity score matching** | | | | | | | | | | | | | | |
|  | **Demographics** | | | | | | | | | | | | | |
|  |  | Cohort | | |  | Mean ± SD | | Patients | | % of Cohort | | | P-Value | Std diff. |
|  |  | 1 2 | | Age | Current Age | 68.8 +/- 7.9 48.3 +/- 8.8 | | 44,315 44,315 | | 100% 100% | | | <0.001 | 2.445 |
|  |  | 1 2 | | AI | Age at Index | 61.9 +/- 7.2 41.2 +/- 7.0 | | 44,315 44,315 | | 100% 100% | | | <0.001 | 2.909 |
|  |  | 1 2 | | F | Female |  | | 0 0 | | 0% 0% | | | -- | -- |
|  |  | 1 2 | | 2054-5 | Black or African American |  | | 2,486 2,674 | | 5.6% 6.0% | | | 0.007 | 0.018 |
|  |  | 1 2 | | M | Male |  | | 44,315 44,315 | | 100% 100% | | | -- | -- |
|  |  | 1 2 | | 2106-3 | White |  | | 24,557 24,449 | | 55.4% 55.2% | | | 0.466 | 0.005 |
|  |  | 1 2 | | 1002-5 | American Indian or Alaska Native |  | | 831 866 | | 1.9% 2.0% | | | 0.391 | 0.006 |
|  |  | 1 2 | | UNK | Unknown Race |  | | 9,113 9,181 | | 20.6% 20.7% | | | 0.573 | 0.004 |
|  |  | 1 2 | | 2076-8 | Native Hawaiian or Other Pacific Islander |  | | 212 201 | | 0.5% 0.5% | | | 0.587 | 0.004 |
|  |  | 1 2 | | UN | Unknown Ethnicity |  | | 15,976 15,900 | | 36.1% 35.9% | | | 0.595 | 0.004 |
|  |  | 1 2 | | 2135-2 | Hispanic or Latino |  | | 5,997 5,920 | | 13.5% 13.4% | | | 0.448 | 0.005 |
|  |  | 1 2 | | 2186-5 | Not Hispanic or Latino |  | | 22,342 22,495 | | 50.4% 50.8% | | | 0.304 | 0.007 |
|  |  | 1 2 | | 2028-9 | Asian |  | | 4,540 4,333 | | 10.2% 9.8% | | | 0.021 | 0.016 |
|  | **Diagnosis** | | | | | | | | | | | | | |
|  |  | Cohort | | |  | Mean ± SD | | Patients | | % of Cohort | | | P-Value | Std diff. |
|  |  | 1 2 | | E08-E13 | Diabetes mellitus |  | | 3,634 3,605 | | 8.2% 8.1% | | | 0.722 | 0.002 |
|  |  | 1 2 | | I30-I52 | Other forms of heart disease (deprecated 2021) |  | | 2,284 2,293 | | 5.2% 5.2% | | | 0.891 | 0.001 |
|  |  | 1 2 | | I20-I25 | Ischemic heart diseases |  | | 828 852 | | 1.9% 1.9% | | | 0.554 | 0.004 |
|  |  | 1 2 | | J40-J4A | Chronic lower respiratory diseases |  | | 2,351 2,345 | | 5.3% 5.3% | | | 0.928 | 0.001 |
|  |  | 1 2 | | I63 | Cerebral infarction |  | | 179 176 | | 0.4% 0.4% | | | 0.873 | 0.001 |
|  |  | 1 2 | | K76.0 | Fatty (change of) liver, not elsewhere classified |  | | 3,718 3,672 | | 8.4% 8.3% | | | 0.576 | 0.004 |
|  |  | 1 2 | | K75 | Other inflammatory liver diseases |  | | 1,645 1,736 | | 3.7% 3.9% | | | 0.111 | 0.011 |
|  |  | 1 2 | | K70 | Alcoholic liver disease |  | | 1,985 2,043 | | 4.5% 4.6% | | | 0.350 | 0.006 |
|  |  | 1 2 | | K71 | Toxic liver disease |  | | 384 418 | | 0.9% 0.9% | | | 0.228 | 0.008 |
|  |  | 1 2 | | B18.2 | Chronic viral hepatitis C |  | | 2,125 2,089 | | 4.8% 4.7% | | | 0.570 | 0.004 |
|  |  | 1 2 | | B18.1 | Chronic viral hepatitis B without delta-agent |  | | 918 956 | | 2.1% 2.2% | | | 0.375 | 0.006 |
|  |  | 1 2 | | C00-D49 | Neoplasms |  | | 3,189 3,214 | | 7.2% 7.3% | | | 0.746 | 0.002 |
|  |  | 1 2 | | I10-I1A | Hypertensive diseases |  | | 6,754 6,555 | | 15.2% 14.8% | | | 0.061 | 0.013 |
|  |  | 1 2 | | I73 | Other peripheral vascular diseases |  | | 148 161 | | 0.3% 0.4% | | | 0.459 | 0.005 |
|  |  | 1 2 | | K75.4 | Autoimmune hepatitis |  | | 243 274 | | 0.5% 0.6% | | | 0.172 | 0.009 |
|  |  | 1 2 | | K75.9 | Inflammatory liver disease, unspecified |  | | 708 752 | | 1.6% 1.7% | | | 0.246 | 0.008 |
|  |  | 1 2 | | K75.81 | Nonalcoholic steatohepatitis (NASH) |  | | 680 696 | | 1.5% 1.6% | | | 0.664 | 0.003 |
|  |  | 1 2 | | K70.9 | Alcoholic liver disease, unspecified |  | | 816 839 | | 1.8% 1.9% | | | 0.568 | 0.004 |
|  |  | 1 2 | | K70.1 | Alcoholic hepatitis |  | | 1,065 1,113 | | 2.4% 2.5% | | | 0.298 | 0.007 |
|  |  | 1 2 | | K70.0 | Alcoholic fatty liver |  | | 252 276 | | 0.6% 0.6% | | | 0.295 | 0.007 |
|  | **Laboratory** | | | | | | | | | | | | | |
|  |  | Cohort | | |  | Mean ± SD | | P-Value | Std diff. | | |  |  |  |
|  |  | 1 2 | | 9050 | Bilirubin.total [Mass/volume] in Serum, Plasma or Blood | 1.5 +/- 2.9 1.9 +/- 4.0 | | <0.001 | 0.119 | | |  |  |  |
|  |  | 1 2 | |  | 0 - 0 mg/dL |  | | 0.726 | 0.002 | | |  |  |  |
|  |  | 1 2 | | 9032 | INR in Plasma or Blood | 1.2 +/- 0.4 1.2 +/- 0.4 | | 0.037 | 0.028 | | |  |  |  |
|  |  | 1 2 | |  | 0 - 0 {INR} |  | | 0.183 | 0.009 | | |  |  |  |
|  |  | 1 2 | | 9024 | Creatinine [Mass/volume] in Serum, Plasma or Blood | 1.1 +/- 4.3 1.0 +/- 2.8 | | <0.001 | 0.045 | | |  |  |  |
|  |  | 1 2 | |  | 0 - 0 mg/dL |  | | 0.619 | 0.003 | | |  |  |  |
|  |  | 1 2 | | 9029 | Sodium [Moles/volume] in Serum, Plasma or Blood | 137.9 +/- 4.2 138.0 +/- 3.9 | | 0.006 | 0.030 | | |  |  |  |
|  |  | 1 2 | |  | 0 - 0 mmol/L |  | | 0.652 | 0.003 | | |  |  |  |
|  |  | 1 2 | | 9045 | Albumin [Mass/volume] in Serum, Plasma or Blood | 3.7 +/- 0.7 3.9 +/- 0.8 | | <0.001 | 0.190 | | |  |  |  |
|  |  | 1 2 | |  | 0 - 0 g/dL |  | | 0.849 | 0.001 | | |  |  |  |
|  |  | 1 2 | | 9083 | BMI | 28.9 +/- 6.4 30.0 +/- 8.0 | | <0.001 | 0.153 | | |  |  |  |
|  |  | 1 2 | |  | 0 - 0 kg/m2 |  | | 0.977 | <0.001 | | |  |  |  |

**Supplementary Table 5 (Baseline Characteristics of Female Patients with Liver Cirrhosis Before and After Propensity Score Matching)**

| **Cohort 1 and cohort 2 patient count before and after propensity score matching** | | | | | | | | | | | | | | |
| --- | --- | --- | --- | --- | --- | --- | --- | --- | --- | --- | --- | --- | --- | --- |
|  | | | Cohort | | | Patient count before matching | | | | | Patient count after matching | | | |
|  | | | 1 - Females 51 to 80 | | | 101,941 | | | | | 27,902 | | | |
|  | | | 2 -Females 21 to 50 | | | 32,446 | | | | | 27,902 | | | |
| **Propensity score density function - Before and after matching (cohort 1 - purple, cohort 2 - green)** | | | | | | | | | | | | | | |
|  |  | | 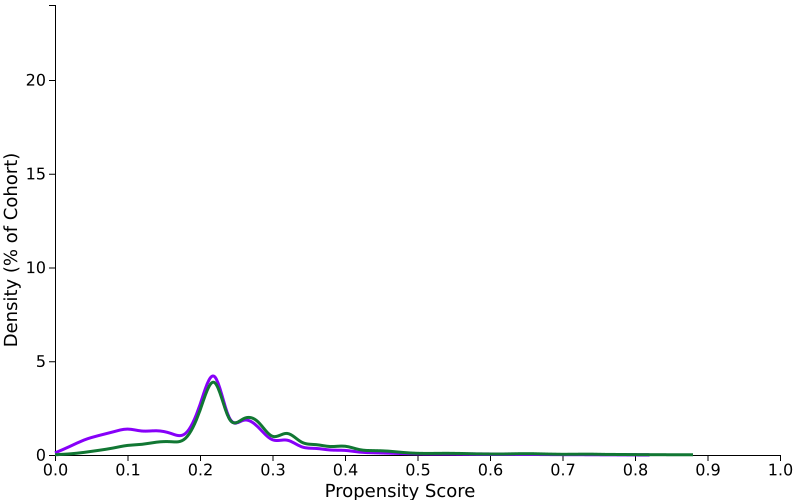 | | | | 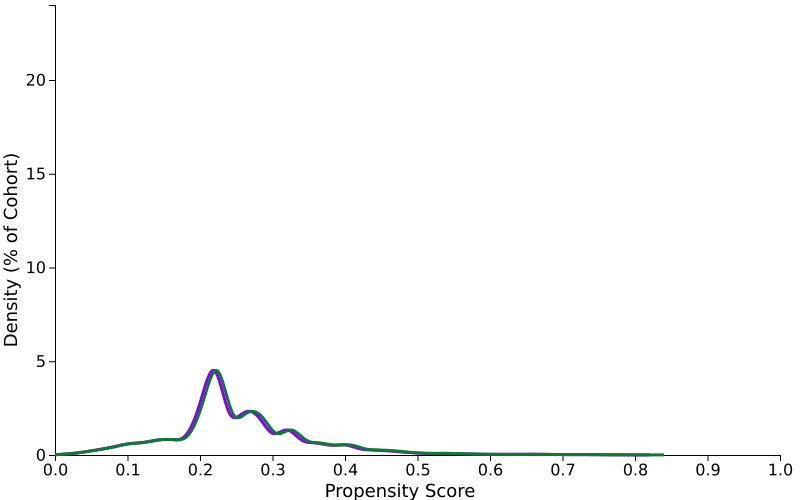 | | | | | | | |
| **Cohort 1 (N = 101,941) and cohort 2 (N = 32,446) characteristics before propensity score matching** | | | | | | | | | | | | | | |
|  | **Demographics** | | | | | | | | | | | | | |
|  |  | Cohort | | |  | Mean ± SD | | Patients | | % of Cohort | | | P-Value | Std diff. |
|  |  | 1 2 | | Age | Current Age | 70.1 +/- 8.1 47.2 +/- 9.1 | | 98,514 28,320 | | 100% 100% | | | <0.001 | 2.654 |
|  |  | 1 2 | | AI | Age at Index | 63.8 +/- 7.6 40.3 +/- 7.6 | | 98,514 28,320 | | 100% 100% | | | <0.001 | 3.089 |
|  |  | 1 2 | | F | Female |  | | 98,514 28,320 | | 100% 100% | | | -- | -- |
|  |  | 1 2 | | 2054-5 | Black or African American |  | | 8,103 2,411 | | 8.2% 8.5% | | | 0.121 | 0.010 |
|  |  | 1 2 | | M | Male |  | | 0 0 | | 0% 0% | | | -- | -- |
|  |  | 1 2 | | 2106-3 | White |  | | 64,573 17,398 | | 65.5% 61.4% | | | <0.001 | 0.086 |
|  |  | 1 2 | | 1002-5 | American Indian or Alaska Native |  | | 946 664 | | 1.0% 2.3% | | | <0.001 | 0.109 |
|  |  | 1 2 | | UNK | Unknown Race |  | | 14,925 4,228 | | 15.2% 14.9% | | | 0.361 | 0.006 |
|  |  | 1 2 | | 2076-8 | Native Hawaiian or Other Pacific Islander |  | | 339 107 | | 0.3% 0.4% | | | 0.398 | 0.006 |
|  |  | 1 2 | | UN | Unknown Ethnicity |  | | 31,243 8,430 | | 31.7% 29.8% | | | <0.001 | 0.042 |
|  |  | 1 2 | | 2135-2 | Hispanic or Latino |  | | 10,341 3,392 | | 10.5% 12.0% | | | <0.001 | 0.047 |
|  |  | 1 2 | | 2186-5 | Not Hispanic or Latino |  | | 56,930 16,498 | | 57.8% 58.3% | | | 0.161 | 0.009 |
|  |  | 1 2 | | 2028-9 | Asian |  | | 5,752 2,121 | | 5.8% 7.5% | | | <0.001 | 0.066 |
|  | **Diagnosis** | | | | | | | | | | | | | |
|  |  | Cohort | | |  | Mean ± SD | | Patients | | % of Cohort | | | P-Value | Std diff. |
|  |  | 1 2 | | E08-E13 | Diabetes mellitus |  | | 19,954 3,203 | | 20.3% 11.3% | | | <0.001 | 0.247 |
|  |  | 1 2 | | I30-I52 | Other forms of heart disease (deprecated 2021) |  | | 11,010 1,974 | | 11.2% 7.0% | | | <0.001 | 0.147 |
|  |  | 1 2 | | I20-I25 | Ischemic heart diseases |  | | 6,917 577 | | 7.0% 2.0% | | | <0.001 | 0.241 |
|  |  | 1 2 | | J40-J4A | Chronic lower respiratory diseases |  | | 13,297 3,148 | | 13.5% 11.1% | | | <0.001 | 0.073 |
|  |  | 1 2 | | I63 | Cerebral infarction |  | | 1,617 163 | | 1.6% 0.6% | | | <0.001 | 0.102 |
|  |  | 1 2 | | K76.0 | Fatty (change of) liver, not elsewhere classified |  | | 12,448 3,806 | | 12.6% 13.4% | | | <0.001 | 0.024 |
|  |  | 1 2 | | K75 | Other inflammatory liver diseases |  | | 7,055 2,161 | | 7.2% 7.6% | | | 0.007 | 0.018 |
|  |  | 1 2 | | K70 | Alcoholic liver disease |  | | 1,636 1,345 | | 1.7% 4.7% | | | <0.001 | 0.176 |
|  |  | 1 2 | | K71 | Toxic liver disease |  | | 1,075 401 | | 1.1% 1.4% | | | <0.001 | 0.029 |
|  |  | 1 2 | | B18.2 | Chronic viral hepatitis C |  | | 6,470 1,137 | | 6.6% 4.0% | | | <0.001 | 0.114 |
|  |  | 1 2 | | B18.1 | Chronic viral hepatitis B without delta-agent |  | | 1,232 489 | | 1.3% 1.7% | | | <0.001 | 0.039 |
|  |  | 1 2 | | C00-D49 | Neoplasms |  | | 21,917 3,919 | | 22.2% 13.8% | | | <0.001 | 0.220 |
|  |  | 1 2 | | I10-I1A | Hypertensive diseases |  | | 31,313 4,463 | | 31.8% 15.8% | | | <0.001 | 0.383 |
|  |  | 1 2 | | I73 | Other peripheral vascular diseases |  | | 2,220 269 | | 2.3% 0.9% | | | <0.001 | 0.104 |
|  |  | 1 2 | | K75.4 | Autoimmune hepatitis |  | | 1,776 685 | | 1.8% 2.4% | | | <0.001 | 0.043 |
|  |  | 1 2 | | K75.9 | Inflammatory liver disease, unspecified |  | | 1,795 703 | | 1.8% 2.5% | | | <0.001 | 0.046 |
|  |  | 1 2 | | K75.81 | Nonalcoholic steatohepatitis (NASH) |  | | 3,870 923 | | 3.9% 3.3% | | | <0.001 | 0.036 |
|  |  | 1 2 | | K70.9 | Alcoholic liver disease, unspecified |  | | 756 452 | | 0.8% 1.6% | | | <0.001 | 0.077 |
|  |  | 1 2 | | K70.1 | Alcoholic hepatitis |  | | 739 919 | | 0.8% 3.2% | | | <0.001 | 0.179 |
|  |  | 1 2 | | K70.0 | Alcoholic fatty liver |  | | 307 174 | | 0.3% 0.6% | | | <0.001 | 0.045 |
|  | **Laboratory** | | | | | | | | | | | | | |
|  |  | Cohort | | |  | Mean ± SD | | P-Value | Std diff. | | |  |  |  |
|  |  | 1 2 | | 9050 | Bilirubin.total [Mass/volume] in Serum, Plasma or Blood | 1.1 +/- 2.3 1.6 +/- 3.7 | | <0.001 | 0.184 | | |  |  |  |
|  |  | 1 2 | |  | 0 - 0 mg/dL |  | | 0.146 | 0.010 | | |  |  |  |
|  |  | 1 2 | | 9032 | INR in Plasma or Blood | 1.2 +/- 0.4 1.2 +/- 0.4 | | <0.001 | 0.055 | | |  |  |  |
|  |  | 1 2 | |  | 0 - 0 {INR} |  | | 0.917 | 0.001 | | |  |  |  |
|  |  | 1 2 | | 9024 | Creatinine [Mass/volume] in Serum, Plasma or Blood | 0.9 +/- 3.3 0.8 +/- 3.1 | | 0.003 | 0.029 | | |  |  |  |
|  |  | 1 2 | |  | 0 - 0 mg/dL |  | | 0.193 | 0.009 | | |  |  |  |
|  |  | 1 2 | | 9029 | Sodium [Moles/volume] in Serum, Plasma or Blood | 138.6 +/- 3.7 138.0 +/- 3.5 | | <0.001 | 0.172 | | |  |  |  |
|  |  | 1 2 | |  | 0 - 0 mmol/L |  | | 0.283 | 0.007 | | |  |  |  |
|  |  | 1 2 | | 9045 | Albumin [Mass/volume] in Serum, Plasma or Blood | 3.8 +/- 0.7 3.8 +/- 0.7 | | 0.009 | 0.026 | | |  |  |  |
|  |  | 1 2 | |  | 0 - 0 g/dL |  | | 0.408 | 0.006 | | |  |  |  |
|  |  | 1 2 | | 9083 | BMI | 30.8 +/- 8.0 31.6 +/- 9.8 | | <0.001 | 0.094 | | |  |  |  |
|  |  | 1 2 | |  | 0 - 0 kg/m2 |  | | 0.328 | 0.007 | | |  |  |  |
| **Cohort 1 (N = 27,902) and cohort 2 (N = 27,902) characteristics after propensity score matching** | | | | | | | | | | | | | | |
|  | **Demographics** | | | | | | | | | | | | | |
|  |  | Cohort | | |  | Mean ± SD | | Patients | | % of Cohort | | | P-Value | Std diff. |
|  |  | 1 2 | | Age | Current Age | 69.6 +/- 8.1 47.2 +/- 9.1 | | 27,902 27,902 | | 100% 100% | | | <0.001 | 2.588 |
|  |  | 1 2 | | AI | Age at Index | 63.1 +/- 7.5 40.4 +/- 7.6 | | 27,902 27,902 | | 100% 100% | | | <0.001 | 3.006 |
|  |  | 1 2 | | F | Female |  | | 27,902 27,902 | | 100% 100% | | | -- | -- |
|  |  | 1 2 | | 2054-5 | Black or African American |  | | 2,263 2,377 | | 8.1% 8.5% | | | 0.080 | 0.015 |
|  |  | 1 2 | | M | Male |  | | 0 0 | | 0% 0% | | | -- | -- |
|  |  | 1 2 | | 2106-3 | White |  | | 17,303 17,175 | | 62.0% 61.6% | | | 0.265 | 0.009 |
|  |  | 1 2 | | 1002-5 | American Indian or Alaska Native |  | | 634 605 | | 2.3% 2.2% | | | 0.405 | 0.007 |
|  |  | 1 2 | | UNK | Unknown Race |  | | 4,148 4,189 | | 14.9% 15.0% | | | 0.626 | 0.004 |
|  |  | 1 2 | | 2076-8 | Native Hawaiian or Other Pacific Islander |  | | 100 106 | | 0.4% 0.4% | | | 0.675 | 0.004 |
|  |  | 1 2 | | UN | Unknown Ethnicity |  | | 8,356 8,339 | | 29.9% 29.9% | | | 0.875 | 0.001 |
|  |  | 1 2 | | 2135-2 | Hispanic or Latino |  | | 3,243 3,340 | | 11.6% 12.0% | | | 0.203 | 0.011 |
|  |  | 1 2 | | 2186-5 | Not Hispanic or Latino |  | | 16,303 16,223 | | 58.4% 58.1% | | | 0.492 | 0.006 |
|  |  | 1 2 | | 2028-9 | Asian |  | | 2,180 2,097 | | 7.8% 7.5% | | | 0.187 | 0.011 |
|  | **Diagnosis** | | | | | | | | | | | | | |
|  |  | Cohort | | |  | Mean ± SD | | Patients | | % of Cohort | | | P-Value | Std diff. |
|  |  | 1 2 | | E08-E13 | Diabetes mellitus |  | | 3,145 3,182 | | 11.3% 11.4% | | | 0.621 | 0.004 |
|  |  | 1 2 | | I30-I52 | Other forms of heart disease (deprecated 2021) |  | | 1,945 1,922 | | 7.0% 6.9% | | | 0.701 | 0.003 |
|  |  | 1 2 | | I20-I25 | Ischemic heart diseases |  | | 576 576 | | 2.1% 2.1% | | | 1 | <0.001 |
|  |  | 1 2 | | J40-J4A | Chronic lower respiratory diseases |  | | 3,153 3,074 | | 11.3% 11.0% | | | 0.288 | 0.009 |
|  |  | 1 2 | | I63 | Cerebral infarction |  | | 149 163 | | 0.5% 0.6% | | | 0.427 | 0.007 |
|  |  | 1 2 | | K76.0 | Fatty (change of) liver, not elsewhere classified |  | | 3,766 3,624 | | 13.5% 13.0% | | | 0.076 | 0.015 |
|  |  | 1 2 | | K75 | Other inflammatory liver diseases |  | | 2,084 2,100 | | 7.5% 7.5% | | | 0.797 | 0.002 |
|  |  | 1 2 | | K70 | Alcoholic liver disease |  | | 992 978 | | 3.6% 3.5% | | | 0.748 | 0.003 |
|  |  | 1 2 | | K71 | Toxic liver disease |  | | 360 378 | | 1.3% 1.4% | | | 0.505 | 0.006 |
|  |  | 1 2 | | B18.2 | Chronic viral hepatitis C |  | | 1,111 1,136 | | 4.0% 4.1% | | | 0.590 | 0.005 |
|  |  | 1 2 | | B18.1 | Chronic viral hepatitis B without delta-agent |  | | 438 470 | | 1.6% 1.7% | | | 0.284 | 0.009 |
|  |  | 1 2 | | C00-D49 | Neoplasms |  | | 3,992 3,887 | | 14.3% 13.9% | | | 0.202 | 0.011 |
|  |  | 1 2 | | I10-I1A | Hypertensive diseases |  | | 4,502 4,438 | | 16.1% 15.9% | | | 0.460 | 0.006 |
|  |  | 1 2 | | I73 | Other peripheral vascular diseases |  | | 264 268 | | 0.9% 1.0% | | | 0.862 | 0.001 |
|  |  | 1 2 | | K75.4 | Autoimmune hepatitis |  | | 665 675 | | 2.4% 2.4% | | | 0.782 | 0.002 |
|  |  | 1 2 | | K75.9 | Inflammatory liver disease, unspecified |  | | 627 657 | | 2.2% 2.4% | | | 0.397 | 0.007 |
|  |  | 1 2 | | K75.81 | Nonalcoholic steatohepatitis (NASH) |  | | 907 915 | | 3.3% 3.3% | | | 0.849 | 0.002 |
|  |  | 1 2 | | K70.9 | Alcoholic liver disease, unspecified |  | | 399 382 | | 1.4% 1.4% | | | 0.540 | 0.005 |
|  |  | 1 2 | | K70.1 | Alcoholic hepatitis |  | | 588 569 | | 2.1% 2.0% | | | 0.572 | 0.005 |
|  |  | 1 2 | | K70.0 | Alcoholic fatty liver |  | | 138 141 | | 0.5% 0.5% | | | 0.857 | 0.002 |
|  | **Laboratory** | | | | | | | | | | | | | |
|  |  | Cohort | | |  | Mean ± SD | | P-Value | Std diff. | | |  |  |  |
|  |  | 1 2 | | 9050 | Bilirubin.total [Mass/volume] in Serum, Plasma or Blood | 1.2 +/- 2.5 1.5 +/- 3.6 | | <0.001 | 0.111 | | |  |  |  |
|  |  | 1 2 | |  | 0 - 0 mg/dL |  | | 0.898 | 0.001 | | |  |  |  |
|  |  | 1 2 | | 9032 | INR in Plasma or Blood | 1.2 +/- 0.5 1.2 +/- 0.4 | | 0.157 | 0.022 | | |  |  |  |
|  |  | 1 2 | |  | 0 - 0 {INR} |  | | 0.825 | 0.002 | | |  |  |  |
|  |  | 1 2 | | 9024 | Creatinine [Mass/volume] in Serum, Plasma or Blood | 0.9 +/- 3.3 0.8 +/- 3.2 | | 0.079 | 0.022 | | |  |  |  |
|  |  | 1 2 | |  | 0 - 0 mg/dL |  | | 0.892 | 0.001 | | |  |  |  |
|  |  | 1 2 | | 9029 | Sodium [Moles/volume] in Serum, Plasma or Blood | 138.7 +/- 3.8 138.0 +/- 3.4 | | <0.001 | 0.170 | | |  |  |  |
|  |  | 1 2 | |  | 0 - 0 mmol/L |  | | 0.980 | <0.001 | | |  |  |  |
|  |  | 1 2 | | 9045 | Albumin [Mass/volume] in Serum, Plasma or Blood | 3.8 +/- 0.7 3.8 +/- 0.7 | | 0.015 | 0.031 | | |  |  |  |
|  |  | 1 2 | |  | 0 - 0 g/dL |  | | 0.959 | <0.001 | | |  |  |  |
|  |  | 1 2 | | 9083 | BMI | 30.3 +/- 7.9 31.8 +/- 9.8 | | <0.001 | 0.167 | | |  |  |  |
|  |  | 1 2 | |  | 0 - 0 kg/m2 |  | | 0.802 | 0.002 | | |  |  |  |
